# Glycemic trends following hypoglossal nerve stimulation in patients with obstructive sleep apnea and type 2 diabetes

**DOI:** 10.1007/s11325-026-03736-6

**Published:** 2026-06-20

**Authors:** Ahsan Ahmed, Shaynie Segal, Marc Gibber

**Affiliations:** 1 Albert Einstein College of Medicine, 1300 Morris Park Ave, Bronx, NY 10461 USA; 2 Department of Otorhinolaryngology – Head and Neck Surgery, Montefiore Medical Center, Bronx, NY USA

**Keywords:** Obstructive sleep apnea, OSA, Hypoglossal nerve stimulation, Type 2 diabetes, Glycemic control

## Abstract

**Purpose:**

To describe glycemic trends following hypoglossal nerve stimulation (HNS) therapy in patients with obstructive sleep apnea (OSA) and type 2 diabetes.

**Methods:**

A retrospective review was conducted of patients with type 2 diabetes who underwent HNS therapy at an academic institution. The study population consisted of 17 diabetic patients who received HNS therapy between Jan 2020 to Oct 2024. Data collected included patient demographics, as well as baseline and post-operative apnea–hypopnea index (AHI) values obtained via polysomnography to assess OSA severity. Diabetes-related parameters included hemoglobin A1c (HbA1c), insulin use, and diabetes medication use. Statistical analyses were performed using IBM SPSS Statistics version 31.0.1.0 with *P* < 0.05 considered statistically significant.

**Results:**

The cohort was 52.9% male with a mean age of 65.3 ± 6.4 years, body mass index of 30.4 ± 3.6 kg/m2, and baseline AHI of 32.4 ± 14.4 events/h. Post-implantation, mean HbA1c decreased from 6.60 to 6.26 (mean change -0.31, *p* = 0.24). Of the 6 patients who were insulin-dependent prior to HNS implantation, 3 (50%) were able to discontinue insulin use during follow-up. Pearson correlation analysis demonstrated an association between AHI improvement and HbA1c improvement (r = -0.69, *p* = 0.027). Greater reductions in medication burden were significantly associated with improvements in both AHI and HbA1c.

**Conclusion:**

In this retrospective cohort, HNS therapy was associated with trends toward lower HbA1c and reduced insulin dependence, although these findings did not reach statistical significance. These results underscore the need for further studies to better understand the role of HNS in improving glycemic control.

## Introduction

Obstructive sleep apnea (OSA) is a highly prevalent disorder characterized by repetitive upper airway collapse during sleep, leading to intermittent hypoxia, sleep fragmentation, and sympathetic activation [1]. In addition to its impacts on sleep, OSA has been identified as an independent risk factor for cardiovascular and metabolic disorders such as hypertension, diabetes, and stroke [2]. While continuous positive airway pressure (CPAP) remains the standard treatment for OSA, adherence and compliance remain significant limiting factors in its effectiveness [3]. Hypoglossal nerve stimulation (HNS) therapy was approved in 2014 and has emerged as a treatment for OSA in patients who are intolerant to continuous positive airway therapy [4]. The therapy works by stimulating the branch of the hypoglossal nerve responsible for tongue protrusion during inspiration, maintaining an open airway [5].

Clinical trials have demonstrated HNS to significantly improve oxygenation and effectively reduce apnea–hypopnea index (AHI), a reference standard for diagnosis of OSA [6]. However, as HNS therapy becomes more widely utilized, understanding its impact on physiological parameters such as metabolic health is increasingly important. Type 2 diabetes is a comorbidity observed in nearly 30% of patients with OSA, significantly higher than the general population when controlling for age and body mass index [7]. Moreover, research has demonstrated that untreated OSA can exacerbate insulin resistance and worsen glycemic control [8]. Understanding how HNS influences diabetes regulation could have significant implications for the management of both conditions [9].

This study aims to explore glycemic trends following HNS therapy by evaluating diabetes-related health outcomes, including hemoglobin A1C (HbA1c), insulin dependency, and diabetes medication usage in patients with moderate to severe OSA. By assessing these parameters before and after HNS implantation, we seek to elucidate whether improvements in sleep-disordered breathing are associated with improved glycemic control. These findings could help establish the role of HNS in the comprehensive care of patients with coexisting OSA and type 2 diabetes.

## Methodology

A retrospective pre-post observational cohort study was conducted of patients with a diagnosis of type 2 diabetes who underwent HNS therapy between June 2020 and October 2024 at a single academic institution. The study included adult patients with moderate to severe OSA, who were intolerant to CPAP with a drug-induced sleep endoscopy (DISE) demonstrating anterior–posterior collapse of the upper airway. Patients with complete concentric collapse at the velum on DISE were excluded. CPAP intolerance was defined as inability to tolerate or maintain adequate adherence to PAP therapy despite attempted use. Patients met institutional criteria for HNS implantation, including age > 18 years, moderate-to-severe OSA (AHI 15–65 events/hour), BMI < 40 kg/m2. Patients who did not undergo in-laboratory full-night polysomnography (PSG) or who lacked postoperative HbA1c measurements were excluded. This study was approved by the Institutional Review Board of Albert Einstein College of Medicine and Montefiore Medical Center (protocol code 2024–16432).

Patient demographics, tobacco and alcohol usage history, baseline apnea–hypopnea index (AHI), and postoperative PSG studies were collected. Final AHI was determined based on in-laboratory, full-night polysomnography conducted after final device titration. Changes in HbA1c, insulin use status, and diabetes medication usage were assessed. The preoperative HbA1c was defined as the most recent value available within 3 months prior to HNS implantation to ensure proximity to intervention. The postoperative HbA1c was defined as the value obtained closest to 6 months following HNS implantation to allow for completion of device titration and stabilization of therapy. Postoperative AHI was determined using the first available in-laboratory full-night polysomnography performed following device titration (mean 240 ± 102 days after implantation). Analyses were performed using all available paired data for each outcome measure. Statistical analyses were performed using IBM SPSS Statistics version 31.0.1.0 (IBM Corp., Armonk, NY). Paired t-tests and Wilcoxon signed-rank tests were used to compare pre- and post-intervention outcomes, while Spearman correlation, Mann–Whitney U, and McNemar tests were used for exploratory and subgroup analyses. Statistical significance was defined as *p* < 0.05.

## Results

Among 80 charts reviewed, 17 met the inclusion criteria for this study and were included. The demographics of our population are summarized in Table 1. The patient population for our study was primarily male (52.9%), with a mean age of 65.3 ± 6.4 years. None were current smokers and 52.9% reported current alcohol usage before implantation. Of the 17 patients with type 2 diabetes, 88.2% (15/17) had diabetes without complication, while 11.8% (2/17) had diabetes with complication.

**Table 1:** Baseline demographic and clinical characteristics of patients undergoing hypoglossal nerve stimulation (*N* = 17)

Characteristic	Value
Age, years	65.3 ± 6.4
Male sex, *n* (%)	9 (52.9)
Race	
White, *n* (%)	9 (52.9)
African American, *n* (%)	5 (29.4)
Other, *n* (%)	3 (17.6)
Smoker, *n* (%)	0 (0)
Alcohol use, *n* (%)	9 (52.9)
Body mass index (BMI), kg/m^2^	30.35 ± 3.64
Apnea–hypopnea index (AHI), events/hour	32.39 ± 14.39
Hemoglobin A1c, %	6.60 ± 1.57
Diabetes without complications, *n* (%)	15 (88.2)
Diabetes with complications, *n* (%)	2 (11.8)
Insulin use at baseline, *n* (%)	6 (35.3)
Device adherence, hours/night*	6.7 ± 2.2
Time to postoperative PSG, days†	240 ± 102

^*^Device adherence derived from stimulation usage data available at follow-up

^†^Mean interval from device implantation to postoperative polysomnography

The mean pre-intervention AHI was 32.4 ± 14.4 events/hour, which decreased to 8.7 ± 5.5 events/hour post-intervention for a mean reduction of 23.98 events/hour, (95% CI −33.07 to −14.90; *p* < 0.001) (Table 2). The average time from device implantation to postoperative sleep study was 240 ± 102 days. Device adherence was high across the cohort, with an average nightly usage of 6.7 h/night. Mean BMI changed from 30.4 ± 3.6 to 29.7 ± 4.1, corresponding to a mean reduction of 0.63 kg/m^2^, (95% CI −1.56 to 0.3, *p* = 0.168). Changes in BMI showed no correlation with changes in HbA1c (r = −0.008, *p* = 0.97). HbA1c decreased from 6.60 ± 1.57 to 6.26 ± 1.18, with a mean reduction of 0.31% (95% CI −0.82 to 0.23; *p* = 0.240). Due to non-normal distribution of HbA1c changes on Shapiro–Wilk testing (*p* < 0.001), a confirmatory Wilcoxon signed-rank analysis was also performed and demonstrated no significant difference (*p* = 0.532). Among the 6 patients who were prescribed insulin therapy at baseline, 3 (50%) were able to discontinue insulin following implantation at 6-month follow-up, 2 (33.3%) required lower daily insulin doses, and 1 (16.7%) remained on an unchanged regimen. Complete insulin discontinuation was not statistically significant on McNemar testing (exact *p* = 0.250).

**Table 2:** Changes in sleep and metabolic outcomes following hypoglossal nerve stimulation

Outcome	Pre-HNS Mean ± SD	Post-HNS Mean ± SD	Mean change (95% CI)	*P* value
AHI (events/hour)	32.39 ± 14.39	8.71 ± 5.50	−23.98 (−33.07 to −14.90)	< 0.001
HbA1c (%)	6.60 ± 1.57	6.26 ± 1.18	−0.31 (−0.85 to 0.23)	0.240
BMI (kg/m^2^)	30.35 ± 3.64	29.72 ± 4.11	−0.63 (−1.56 to 0.30)	0.168

**Fig. 1:**
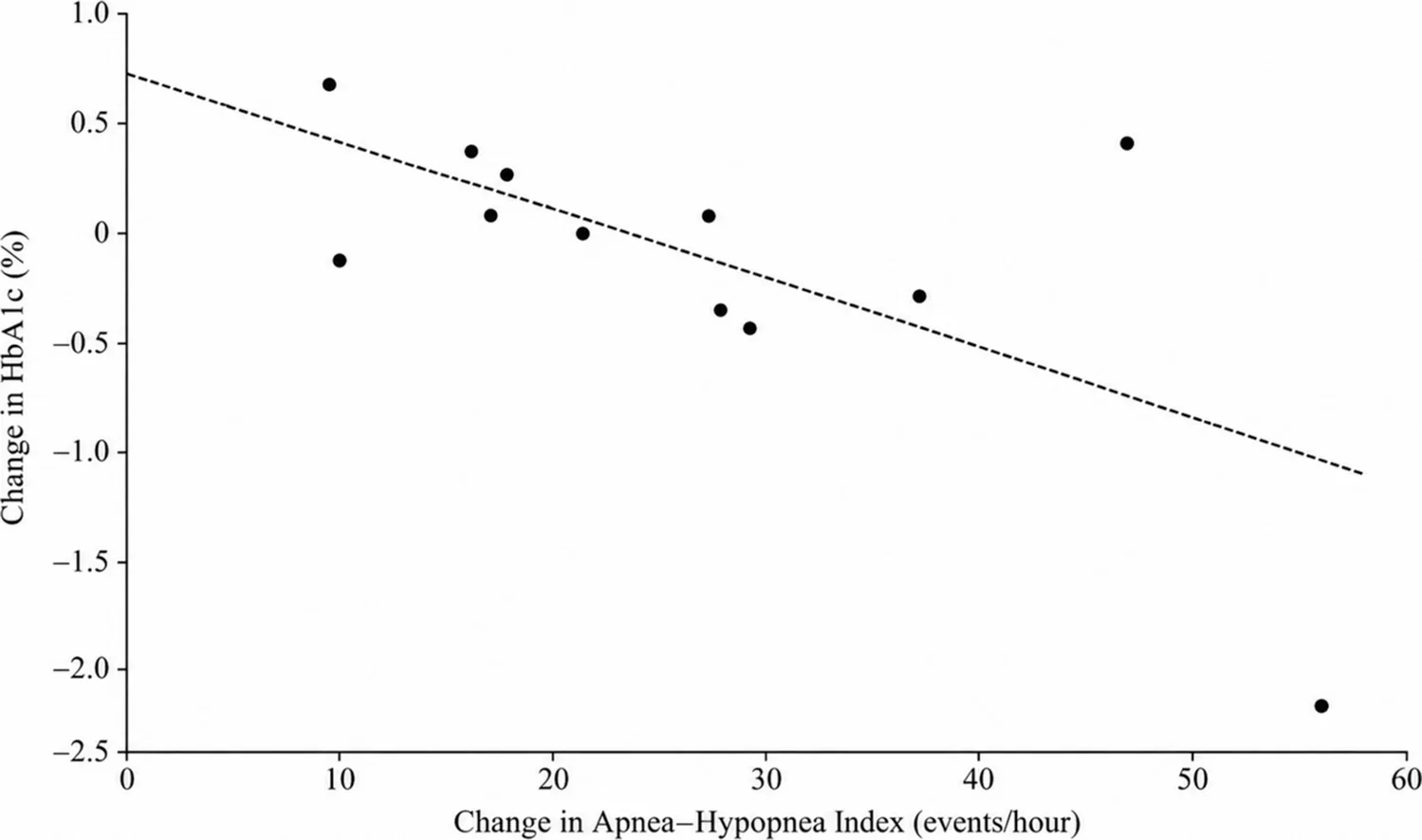
Relationship between change in apnea–hypopnea index and change in hemoglobin A1c following hypoglossal nerve stimulation

The overall distribution of diabetes medication did not significantly change following implantation (Wilcoxon signed-rank *p* = 0.564). The number of patients prescribed GLP-1 agonists increased from 3 to 4, while those prescribed SGLT2 inhibitors increased from 2 to 4. The distribution of diabetes medication and total medication burden are summarized in Tables 3 and 4. Greater reductions in diabetes medication burden were significantly associated with greater improvements in both AHI (Spearman’s rho = 0.682, *p* = 0.010) and HbA1c (Spearman’s rho = 0.693, *p* = 0.009) (Fig. 1).

**Table 3:** Diabetes medication classes (Metformin, GLP-1, SGLT2, etc.)

Medication class	Pre-HNS *n* (%)	Post-HNS *n* (%)
Metformin	8 (47.1)	7 (41.2)
Sitagliptin	3 (17.6)	3 (17.6)
GLP-1 Agonists (Liraglutide, Semaglutide, Exenatide)	3 (17.6)	4 (23.5)
SGLT2 Inhibitors (Empagliflozin, Dapagliflozin)	2 (11.8)	4 (23.5)
Insulin	6 (35.3)	3 (17.6)

**Table 4:** Number of diabetes medication

Number of diabetes medications	Pre-HNS *n* (%)	Post-HNS *n* (%)
0	6 (35.3)	6 (35.3)
1–2	10 (58.8)	8 (47.1)
≥ 3	1 (5.9)	3 (17.6)

Pearson correlation analysis demonstrated a moderate association between AHI reduction and HbA1c improvement (r = 0.695, *p* = 0.026); however, this association was not significant using Spearman rank correlation (rho = 0.462, *p* = 0.179) (Fig. 1).

## Discussion

OSA is a highly prevalent disorder, approximated to affect almost one billion adults worldwide, with estimates that only 40% of those with OSA have been diagnosed [10, 11]. OSA is strongly associated with a range of comorbidities, including hypertension, stroke, cardiac arrhythmias, type 2 diabetes and reflux [12]. During sleep, OSA patients experience recurrent airway obstruction, triggering intermittent hypoxia, which is accompanied by a chronic sympathetic nervous system activation [1]. Evidence suggests that this sympathetic overdrive leads to higher heart rate, higher blood pressure, and increased cardiovascular risk than healthy controls [13]. As can be anticipated, these patients are also at an increased risk for early all-cause mortality [14].

CPAP is the gold standard treatment for all severity levels of OSA. However, it is often poorly tolerated by patients with suboptimal adherence [15]. Research has demonstrated that 40% of patients have suboptimal usage of CPAP and 20–30% of patients discontinue CPAP usage during their first year of treatment [16]. Since FDA approval in 2014, HNS has been widely used in select patients with moderate-to-severe OSA who do not benefit from positive airway pressure [17].

Beyond symptomatic relief, treating OSA has significant implications for mitigating comorbidities such as diabetes [18]. The current consensus in the medical literature is that there is a well-established association between diabetes and OSA [18]. OSA severity was shown to be positively associated with the incidence of type 2 diabetes, independent of BMI or age [7]. However, there is minimal research evaluating how HNS therapy influences glycemic control. To our knowledge, this retrospective review is only the second study to specifically examine glycemic outcomes following HNS therapy [19]. Among the six patients receiving insulin therapy at baseline, three discontinued insulin therapy entirely, two required lower insulin doses during follow-up, and one remained on an unchanged regimen. Although these findings did not reach statistical significance, they may suggest a trend toward improved glycemic management following HNS therapy. While these findings are preliminary, incremental decreases in HbA1c have been shown to offer substantial health benefits. A meta-analysis by Giugliano et al., reported that reductions of ≥ 0.3% in HbA1c were significantly associated with lower rates of major adverse cardiovascular events [20]. Similarly, Fralick et al., reported that each 0.5% decrease in HbA1c was correlated with improved cardiovascular outcomes, with 0.3% remaining clinically relevant [21].

Interestingly, BMI changed minimally in our cohort and was not correlated with change in HbA1c, suggesting that observed glycemic trends were not explained by weight change alone. Although the overall distribution of diabetes medications did not significantly change following implantation, greater reductions in diabetes medication burden were significantly associated with improvements in both AHI and HbA1c. This finding may suggest that patients demonstrating greater therapeutic response to HNS also exhibit more favorable diabetes-related outcomes. Additionally, Pearson correlation analysis demonstrated an association between AHI reduction and HbA1c improvement; however, this finding was not significant using Spearman rank correlation, and should therefore be interpreted cautiously. Collectively, these findings suggest the possibility that improvements in sleep-disordered breathing may be associated with favorable metabolic trends. These observations are supported by randomized controlled trials demonstrating associations between improvements in AHI and glycemic measures following CPAP therapy [22].

Our findings are very similar to those of a previous HNS study with 14 patients, which demonstrated a mean HbA1c reduction from 6.67 to 6.16 (Δ = –0.51; *p* = 0.16) [19]. Only two patients in the cohort were on diabetic medications, and insulin dependency was not analyzed. Similarly, in randomized controlled trials of CPAP efficacy on diabetes control in patients with OSA, improvements in insulin sensitivity and glycemic control have been reported with long-term adherence [23]. One RCT found that CPAP led to no significant HbA1c reduction after 3 months but a statistically significant reduction after 6 months of adherence (0.510, *p* = 0.002) [22].

Several mechanisms of a relationship between diabetes and OSA have been proposed. One proposed pathway is that in patients with treated OSA, the elimination of intermittent hypoxia might improve the regulation of carbohydrate metabolism as hypoxia is accepted as one of the main determinants of HbA1c in these patients [24]. The effect of hypoxia on glycemic control might be mediated by hypoxia-inducible-factor-1, which triggers the expression of specific genes in the presence of low oxygen levels [25]. Henceforth, in diabetic rats, an increase in hypoxia-inducible-factor-1 expression by pancreatic beta cells, which inhibits glucose transport and perpetuates a state of insulin resistance, has been reported [25]. Another mechanism proposed is that increased sympathetic nervous system activation leads to an increase in glycogenolysis and gluconeogenesis in the liver, a decrease of insulin release in the pancreas, and increased lipolysis in adipose tissue [26].

While CPAP therapy has shown efficacy in improving metabolic outcomes, not all findings are consistent. Three meta-analyses evaluating RCTs on the effect of CPAP therapy on glycemic control in OSA patients with type 2 diabetes yielded different findings. The most recent meta-analysis showed significant reductions in HbA1c (−0.32%, *p* = 0.029) while the other two meta-analysis showed no effects of CPAP treatment on glycemic control [27, 28]. A cause of variation is that effect of CPAP treatment is highly variable based on patient adherence to treatment. Research has demonstrated that OSA recurs within 1–2 nights after discontinuation of CPAP, which is associated with a short-term increase of sympathetic activation [29]. If patients did not use their CPAP before glycemic control assessment, this might have affected their parameters. Based on the variability within CPAP trials on adherence, we sought to determine whether there was any correlation between changes in HbA1c level based on device adherence. However, device adherence remained high within our cohort with an average of 6.7 h/night usage and did not demonstrate any correlation with changes in HbA1c.

The recent FDA approval of tirzepatide, a dual GLP-1/GIP agonist, for the treatment of moderate to severe OSA in adults with obesity highlights an important consideration when interpreting our findings [30]. In our cohort, the number of patients prescribed GLP-1 receptor agonists increased during follow-up (from three to four). Given evidence that GLP-1 agonists may improve both OSA severity and glycemic control, their use may represent a confounding factor in attributing metabolic improvements to HNS therapy. While our study was not powered to perform subgroup analysis, this overlap underscores the need for further investigations to fully evaluate the interaction between GLP-1 agonists and HNS outcomes.

Our study is one of the first to assess diabetes-specific outcomes following OSA surgery. A strength of our study is that the duration of the follow-up was > 6 months, allowing sufficient time to observe metabolic changes. Additionally, we incorporated objective clinical metrics such as Hemoglobin A1c, insulin use status, and medication count to provide a multidimensional evaluation of glycemic control.

Limitations of our study include the sample size of 17 patients, limiting our statistical power and the ability to perform more detailed adjusted analyses. We were unable to account for potential confounders such as medication adjustments, dietary habits, or physical activity. Changes in diabetic medications, including increased use of GLP-1 receptor agonists, may have independently affected HbA1c levels. As such, the observed associations cannot be attributed solely to HNS therapy. The cohort had relatively mild baseline diabetes and obesity, which may limit generalizability to patients with more severe metabolic disease. Additionally, postoperative HbA1c and PSG measurements were not necessarily obtained during identical follow-up windows. Given the retrospective exploratory design and limited number of eligible patients, an a priori sample size calculation was not performed. Larger prospective controlled studies are needed to further characterize glycemic trends following HNS therapy and determine their potential clinical significance.

## Conclusion

In conclusion, among patients with type 2 diabetes and OSA, HNS treatment was associated with nonsignificant trends toward lower HbA1c and reduced insulin use. Improvements in diabetes medication burden were significantly associated with improvements in both AHI and HbA1c, suggesting a potential relationship between treatment response and glycemic outcomes. Additionally, exploratory analyses suggested a potential association between improvements in OSA severity and glycemic measures. Larger prospective controlled studies with comparable patient cohorts are needed to further characterize the relationship between HNS therapy and glycemic control and to determine which patient populations can most benefit from this therapy.

## Data Availability

The datasets generated and/or analyzed during the current study are not publicly available due to patient confidentiality and institutional policy but are available from the corresponding author on reasonable request.

## References

[1] Abbasi A, Gupta SS, Sabharwal N et al (2021) A comprehensive review of obstructive sleep apnea. Sleep Sci 14(2):142–154. 10.5935/1984-0063.20200056 34381578 10.5935/1984-0063.20200056 PMC8340897

[2] Qian Y, Dharmage SC, Hamilton GS et al (2023) Longitudinal risk factors for obstructive sleep apnea: a systematic review. Sleep Med Rev 71:101838. 10.1016/j.smrv.2023.101838 37639973 10.1016/j.smrv.2023.101838

[3] Kim DH, Kim SW, Han JS et al (2024) Comparative effectiveness of hypoglossal nerve stimulation and alternative treatments for obstructive sleep apnea: a systematic review and meta-analysis. J Sleep Res 33(3):e14017. 10.1111/jsr.14017 37661785 10.1111/jsr.14017

[4] Strollo PJ Jr, Soose RJ, Maurer JT et al (2014) Upper-airway stimulation for obstructive sleep apnea. N Engl J Med 370(2):139–149. 10.1056/NEJMoa1308659 24401051 10.1056/NEJMoa1308659

[5] Olson MD, Junna MR (2021) Hypoglossal nerve stimulation therapy for the treatment of obstructive sleep apnea. Neurotherapeutics 18(1):91–99. 10.1007/s13311-021-01012-x 33559036 10.1007/s13311-021-01012-x PMC8116425

[6] Medical Advisory Secretariat (2006) Polysomnography in patients with obstructive sleep apnea: an evidence-based analysis. Ont Health Technol Assess Ser 6(13):1–38 PMC3379160 23074483

[7] Mahmood K, Akhter N, Eldeirawi K et al (2009) Prevalence of type 2 diabetes in patients with obstructive sleep apnea in a multi-ethnic sample. J Clin Sleep Med 5(3):215–221 19960641 PMC2699165

[8] Aronsohn RS, Whitmore H, Van Cauter E, Tasali E (2010) Impact of untreated obstructive sleep apnea on glucose control in type 2 diabetes. Am J Respir Crit Care Med 181(5):507–513. 10.1164/rccm.200909-1423OC 20019340 10.1164/rccm.200909-1423OC PMC2830401

[9] Valensi P, Benmohammed K, Zerguine M (2025) Bidirectional interplay of sleep apnea syndrome and cardio-vascular disorders in diabetes. Diabetes Res Clin Pract 220:111984. 10.1016/j.diabres.2024.111984 39761874 10.1016/j.diabres.2024.111984

[10] Benjafield AV, Ayas NT, Eastwood PR et al (2019) Estimation of the global prevalence and burden of obstructive sleep apnoea: a literature-based analysis. Lancet Respir Med 7(8):687–698. 10.1016/S2213-2600(19)30198-5 31300334 10.1016/S2213-2600(19)30198-5 PMC7007763

[11] Young T, Palta M, Dempsey J, Peppard PE, Nieto FJ, Hla KM (2009) Burden of sleep apnea: rationale, design, and major findings of the Wisconsin sleep cohort study. WMJ 108(5):246–249 19743755 PMC2858234

[12] Gottlieb DJ, Yenokyan G, Newman AB et al (2010) Prospective study of obstructive sleep apnea and incident coronary heart disease and heart failure: the sleep heart health study. Circulation 122(4):352–360. 10.1161/CIRCULATIONAHA.109.901801 20625114 10.1161/CIRCULATIONAHA.109.901801 PMC3117288

[13] Narkiewicz K, Montano N, Cogliati C, van de Borne PJ, Dyken ME, Somers VK (1998) Altered cardiovascular variability in obstructive sleep apnea. Circulation 98(11):1071–1077. 10.1161/01.cir.98.11.1071 9736593 10.1161/01.cir.98.11.1071

[14] Rich J, Raviv A, Raviv N, Brietzke SE (2011) An epidemiologic study of snoring and all-cause mortality. Otolaryngol Head Neck Surg 145(2):341–346. 10.1177/0194599811402475 21493281 10.1177/0194599811402475

[15] Qaseem A, Holty JE, Owens DK et al (2013) Management of obstructive sleep apnea in adults: a clinical practice guideline from the American College of Physicians. Ann Intern Med 159(7):471–483. 10.7326/0003-4819-159-7-201310010-00704 24061345 10.7326/0003-4819-159-7-201310010-00704

[16] Lévy P, Kohler M, McNicholas WT et al (2015) Obstructive sleep apnoea syndrome. Nat Rev Dis Prim 1:15015. 10.1038/nrdp.2015.15. (**Published 2015 Jun 25**) 27188535 10.1038/nrdp.2015.15

[17] Suurna MV, Klasner M (2024) Neurostimulation for obstructive sleep apnea. Otolaryngol Clin North Am 57(3):457–465. 10.1016/j.otc.2024.02.003 38521724 10.1016/j.otc.2024.02.003

[18] Reutrakul S, Mokhlesi B (2017) Obstructive sleep apnea and diabetes: a state of the art review. Chest 152(5):1070–1086. 10.1016/j.chest.2017.05.009 28527878 10.1016/j.chest.2017.05.009 PMC5812754

[19] Freeman CG, Durgham R, Ren E et al (n.d.) The impact of hypoglossal nerve stimulation on secondary health outcomes. Ear Nose Throat J. 10.1177/01455613241235538. (Published online February 29, 2024) 10.1177/01455613241235538 38424691

[20] Giugliano D, Chiodini P, Maiorino MI, Bellastella G, Esposito K (2019) Cardiovascular outcome trials and major cardiovascular events: does glucose matter? A systematic review with meta-analysis. J Endocrinol Invest 42(10):1165–1169. 10.1007/s40618-019-01047-0 30955180 10.1007/s40618-019-01047-0

[21] Fralick M, Colacci M, Odutayo A, Siemieniuk R, Glynn RJ (2020) Lowering of hemoglobin A1C and risk of cardiovascular outcomes and all-cause mortality, a meta-regression analysis. J Diabetes Complicat 34(11):107704. 10.1016/j.jdiacomp.2020.107704 10.1016/j.jdiacomp.2020.107704 32888788

[22] Martínez-Cerón E, Barquiel B, Bezos AM, Casitas R, Galera R, García-Benito C, Hernanz A, Alonso-Fernández A, Garcia-Rio F (2016) Effect of continuous positive airway pressure on glycemic control in patients with obstructive sleep apnea and type 2 diabetes. A randomized clinical trial. Am J Respir Crit Care Med 194(4):476–85. 10.1164/rccm.201510-1942OC 26910598 10.1164/rccm.201510-1942OC

[23] Herth J, Sievi NA, Schmidt F, Kohler M (2023) Effects of continuous positive airway pressure therapy on glucose metabolism in patients with obstructive sleep apnoea and type 2 diabetes: a systematic review and meta-analysis. Eur Respir Rev 32(169):230083. 10.1183/16000617.0083-2023 37673425 10.1183/16000617.0083-2023 PMC10481331

[24] Cantley J, Grey ST, Maxwell PH, Withers DJ (2010) The hypoxia response pathway and β-cell function. Diabetes Obes Metab 12(Suppl 2):159–167. 10.1111/j.1463-1326.2010.01276.x 21029313 10.1111/j.1463-1326.2010.01276.x

[25] Bensellam M, Duvillié B, Rybachuk G, Laybutt DR, Magnan C, Guiot Y, Pouysségur J, Jonas JC (2012) Glucose-induced O₂ consumption activates hypoxia inducible factors 1 and 2 in rat insulin-secreting pancreatic beta-cells. PLoS One 7(1):e29807. 10.1371/journal.pone.0029807 22235342 10.1371/journal.pone.0029807 PMC3250482

[26] Carnagarin R, Matthews VB, Herat LY, Ho JK, Schlaich MP (2018) Autonomic regulation of glucose homeostasis: a specific role for sympathetic nervous system activation. Curr Diab Rep 18(11):107. 10.1007/s11892-018-1069-2 30232652 10.1007/s11892-018-1069-2

[27] Zhu B, Ma C, Chaiard J, Shi C (2018) Effect of continuous positive airway pressure on glucose metabolism in adults with type 2 diabetes: a systematic review and meta-analysis of randomized controlled trials. Sleep Breath 22(2):287–295. 10.1007/s11325-017-1554-x 28812180 10.1007/s11325-017-1554-x

[28] Labarca G, Reyes T, Jorquera J, Dreyse J, Drake L (2018) CPAP in patients with obstructive sleep apnea and type 2 diabetes mellitus: systematic review and meta-analysis. Clin Respir J 12(8):2361–2368. 10.1111/crj.12915 30073792 10.1111/crj.12915

[29] Schwarz EI, Stradling JR, Kohler M (2018) Physiological consequences of CPAP therapy withdrawal in patients with obstructive sleep apnoea-an opportunity for an efficient experimental model. J Thorac Dis 10(Suppl 1):S24–S32. 10.21037/jtd.2017.12.142 29445525 10.21037/jtd.2017.12.142 PMC5803046

[30] Malhotra A, Grunstein RR, Fietze I et al (2024) Tirzepatide for the treatment of obstructive sleep apnea and obesity. N Engl J Med 391(13):1193–1205. 10.1056/NEJMoa2404881 38912654 10.1056/NEJMoa2404881 PMC11598664

